# Prevalence and correlates of common mental disorders among participants of the Uganda Genome Resource: Opportunities for psychiatric genetics research

**DOI:** 10.1038/s41380-024-02665-8

**Published:** 2024-07-14

**Authors:** Allan Kalungi, Eugene Kinyanda, Dickens Howard Akena, Bizu Gelaye, Wilber Ssembajjwe, Richard Steven Mpango, Terry Ongaria, Joseph Mugisha, Ronald Makanga, Ayoub Kakande, Beatrice Kimono, Philip Amanyire, Fred Kirumira, Cathryn M. Lewis, Andrew M. McIntosh, Karoline Kuchenbaecker, Moffat Nyirenda, Pontiano Kaleebu, Segun Fatumo

**Affiliations:** 1 The African Computational Genomics (TACG) Research Group, Medical Research Council/ Uganda Virus Research Institute & London School of Hygiene and Tropical Medicine (MRC/UVRI & LSHTM) Uganda Research Unit, Entebbe, Uganda; 2 Department of Medical Biochemistry, College of Health Sciences, Makerere University, Kampala, Uganda; 3 The Department of Non-communicable Diseases Epidemiology, London School of Hygiene and Tropical Medicine London, London, UK; 4 Mental Health Section, Medical Research Council/ Uganda Virus Research Institute & London School of Hygiene and Tropical Medicine (MRC/UVRI & LSHTM) Uganda Research Unit, Entebbe, Uganda; 5 Department of Psychiatry, College of Health Sciences, Makerere University, Kampala, Uganda; 6 Department of Epidemiology, Harvard T.H. Chan School of Public Health, 677 Huntington Ave Room 505F, Boston, MA 02115 USA; 7 The Chester M. Pierce, MD Division of Global Psychiatry, Massachusetts General Hospital, Boston, MA USA; 8 Department of Psychiatry, Harvard Medical School, Boston, MA USA; 9 Medical Research Council/ Uganda Virus Research Institute & London School of Hygiene and Tropical Medicine (MRC/UVRI & LSHTM) Uganda Research Unit, Entebbe, Uganda; 10 Social, Genetic and Developmental Psychiatry Centre, Institute of Psychiatry, Psychology & Neuroscience, King’s College London, de Crespigny Park, London, SE5 8AF UK; 11 Division of Psychiatry, University of Edinburgh, Royal Edinburgh Hospital, Edinburgh, UK; 12 Division of Psychiatry, UCL Genetics Institute, University College London, London, UK; 13 Precision Healthcare University Research Institute, Queen Mary University of London, London, UK

**Keywords:** Psychiatric disorders, ADHD, Genetics

## Abstract

Genetics research has potential to alleviate the burden of mental disorders in low- and middle-income-countries through identification of new mechanistic pathways which can lead to efficacious drugs or new drug targets. However, there is currently limited genetics data from Africa. The Uganda Genome Resource provides opportunity for psychiatric genetics research among underrepresented people from Africa. We aimed at determining the prevalence and correlates of major depressive disorder (MDD), suicidality, post-traumatic stress disorder (PTSD), alcohol abuse, generalised anxiety disorder (GAD) and probable attention-deficit hyperactivity disorder (ADHD) among participants of the Uganda Genome Resource. Standardised tools assessed for each mental disorder. Prevalence of each disorder was calculated with 95% confidence intervals. Multivariate logistic regression models evaluated the association between each mental disorder and associated demographic and clinical factors. Among 985 participants, prevalence of the disorders were: current MDD 19.3%, life-time MDD 23.3%, suicidality 10.6%, PTSD 3.1%, alcohol abuse 5.7%, GAD 12.9% and probable ADHD 9.2%. This is the first study to determine the prevalence of probable ADHD among adult Ugandans from a general population. We found significant association between sex and alcohol abuse (adjusted odds ratio [AOR] = 0.26 [0.14,0.45], p < 0.001) and GAD (AOR = 1.78 [1.09,2.49], p = 0.019) respectively. We also found significant association between body mass index and suicidality (AOR = 0.85 [0.73,0.99], p = 0.041), alcohol abuse (AOR = 0.86 [0.78,0.94], p = 0.003) and GAD (AOR = 0.93 [0.87,0.98], p = 0.008) respectively. We also found a significant association between high blood pressure and life-time MDD (AOR = 2.87 [1.08,7.66], p = 0.035) and probable ADHD (AOR = 1.99 [1.00,3.97], p = 0.050) respectively. We also found a statistically significant association between tobacco smoking and alcohol abuse (AOR = 3.2 [1.56,6.67], p = 0.002). We also found ever been married to be a risk factor for probable ADHD (AOR = 2.12 [0.88,5.14], p = 0.049). The Uganda Genome Resource presents opportunity for psychiatric genetics research among underrepresented people from Africa.

## Introduction

Mental disorders have persisted among the top ten leading causes of disease burden worldwide, with no evidence of global reduction in the burden since 1990 [1]. It is estimated that 1 in every 8 people in the world lives with a mental disorder [2]. Mental disorders account for 4.9% of the global disability-adjusted life-years and 14.6% of the global years lived with disability [1]. Some of the common mental disorders include major depressive disorder (MDD), generalized anxiety disorder (GAD), attention-deficit hyperactivity disorder (ADHD), suicidality and post-traumatic stress disorder (PTSD).

The global age-standardised prevalence of any mental disorder has been estimated at 12.3% [1] while the individual global prevalence for anxiety disorders, depressive disorders and attention-deficit hyperactivity disorder (ADHD) have been estimated at 3.8%, 3.4% and 1.1% respectively, accounting for point, 12 month and lifetime prevalence using pooled prevalence ratios [1].

Over 80% of the burden of mental disorders pertains to low- and middle-income-countries (LMIC) [3] where the treatment gap for psychiatric disorders approaches 90% [4]. In Sub-Saharan Africa (SSA) an age-standardised prevalence of 13.4% has been estimated for any mental disorder [1] while prevalence of 4.5%, 3.5% and 0.6% has been reported for depressive disorders, anxiety disorders and ADHD respectively. The age-standardised prevalence of depressive disorders is higher in SSA than any other region globally [1].

In Uganda, the prevalence of any mental disorder has been estimated at 24.2% (95% C.I 19.8%–28.6%) among adults [5]. For depression, a pooled prevalence of 30.2% has been determined by a systematic review and a meta-analysis among heterogeneous samples [6] while prevalence estimates of 4.2–29.3% have been determined among general populations from various study sites in Uganda [7–11]. For anxiety disorders, a prevalence of 22.2% has been reported among adults in Uganda [5]. Combined together, depression and anxiety disorders have been reported to affect approximately one in four persons in Uganda [5].

Mental disorders are a huge public health problem with marked consequences for society. They lead to severe distress and functional impairment in several domains including social and work environments [12] and those which set on early in life have been strongly associated with truncated education attainment [13]. There is currently no cure for most mental illnesses. However, there are evidenced psychological and pharmacological treatments which have been found to effectively minimize the symptoms so as to allow the individual to function in work, school, or social environments. These are described for the various mental disorders below;

### Major depressive disorder

Can be managed with various treatment modalities, including pharmacological, psychotherapeutic, interventional, and lifestyle modification [14]. Initial treatment of MDD involves medications and/or psychotherapy, with combination therapy proving more effective than either alone [15, 16]. Antidepressants, classified as selective serotonin reuptake inhibitors (SSRIs), serotonin-norepinephrine reuptake inhibitors (SNRIs), serotonin modulators, tricyclic antidepressants (TCAs) and monoamine oxidase inhibitors (MAOIs) and atypical antidepressants are commonly used [14]. SSRIs, such as fluoxetine and sertraline, are first-line choices, and they acting by increasing serotonin activity [17]. SNRIs, like venlafaxine, target non-responders or those with comorbid pain disorders. Other classes include serotonin modulators, TCAs, MAOIs, and atypical antidepressants like bupropion, agomelatine, and mirtazapine, each with distinct mechanisms [18–20].

For psychotherapeutic treatment, this is normally considered for mild to moderate MDD. It emphasizes cognitive-behavioural therapy (CBT) and interpersonal therapy (IPT), supplemented by supportive therapy and psychoeducation [21]. Additional treatments comprise electroconvulsive therapy, proven highly efficacious for severe MDD [22], and physical exercise [23].

### Suicidality

The most widely used psychotherapeutic interventions for suicidality are dialectical behavioural therapy and CBT while other innovative interventions like group therapies and internet-based therapies have been tried but these require further study [24]. For pharmacotherapy, a strong evidence of both lithium and ketamine in reducing the risk of suicide has been documented [25].

### Post-traumatic stress disorder

Trauma focused psychotherapies are currently the gold standard in treatment of trauma-associated symptoms of PTSD and they include cognitive processing therapy, prolonged exposure therapy and eye movement, desensitization and restructuring therapy [26]. For pharmacotherapy, SSRIs and SNRIs such as fluoxetine, paroxetine, sertraline and venlafaxine have been reported to reduce PTSD symptoms when administered at appropriate doses [26].

### Alcohol abuse

In pharmacotherapeutic management, naltrexone is the first-line treatment option for alcohol abuse owing to its preferable dosing schedule and ability for treatment to be initiated while the individual is still drinking [27]. Acamprosate has been described as an alternative to naltrexone in patients with contraindications to naltrexone [27]. For patients who are contraindicated or those who do not respond to these two, the next choices are disulfiram or topiramate [27]. Psychotherapeutic treatments for alcohol abuse include motivational enhancement therapy and the twelve-step programs [28, 29]. However, a combination of CBT and pharmacotherapy has been reported to be more efficacious in treating alcohol abuse as compared with usual care and pharmacotherapy [30].

### Generalized anxiety disorder

CBT is the psychotherapeutic treatment with the highest evidence for treatment of GAD [31]. However internet therapies have also been proposed where CBT may not be easily available [31]. Pharmacotherapeutic treatment involves using SSRIs (such as escitalopram, paroxetine and sertraline) and SNRIs (such as doluxetine and venlafaxine) as first-line drugs for GAD [31]. Other evidenced drugs in the treatment of GAD include buspirone and benzodiazepines [31].

### Attention-deficit hyperactivity disorder

ADHD management involves medical and psychological approaches. Medical options include stimulants (e.g., amphetamines, methylphenidates) or non-stimulants (e.g.,TCAs, non-tricyclic antidepressants, norepinephrine reuptake inhibitors, alpha-2 noradrenergic agonists). Stimulants work by enhancing neurotransmission but their misuse is a concern [32]. Psychological treatments like CBT, mindfulness, dialectical behavior therapy, and neurofeedback complement medications, though reported less effective in symptom alleviation. They prove useful in addressing residual problems post-medication use [32–37].

Mental disorders are complex disorders which arise from complex systems which include several factors such as psychological, genetic, biological, socioeconomic and environmental factors [38]. Mental disorders are heritable and heritability estimates based on sibling data have been reported to vary from 30% for MDD to 80% for ADHD [39]. Genetics studies have potential to identify new mechanistic pathways for mental disorders, which can, in turn lead to new drugs or drug targets for several mental disorders. Recent genome-wide association studies have provided insights into the genetic architecture of several mental disorders like MDD [40], suicidality [41], PTSD [42], schizophrenia [43], alcohol abuse [44], GAD [45] and ADHD [46].

Despite the genetic nature of several mental disorders being illuminated, there is limited genetics data from Africa. There is an urgent need to include people on the Africa continent (continental Africans) in global psychiatric genetics research if they (continental Africans) are to benefit from recent psychiatric genetics discoveries. The Uganda Genome Resource [47] provides an opportunity for psychiatric genetics research among people from Uganda. Briefly, the Uganda Genome Resource comprises genotype data on ∼5000 and whole-genome sequence data on ∼2000 Ugandan individuals from 10 ethno-linguistic groups who are attending an open general population cohort (GPC) in south-western Uganda [47, 48]. This cohort has contributed to several scientific discoveries in Uganda and worldwide [47] and is run by the MRC/UVRI and LSHTM Uganda Research Unit and the current study determined the prevalence and correlates of several mental disorders among participants who are attending the cohort and are also part of the Uganda Genome Resource.

## Methods

### Study design

This study was undertaken within the GPC of MRC/UVRI & LSHTM Uganda Research Unit. The GPC is an active cohort of approximately 22,000 participants within 25 villages drawn from a sub-county in Kalungu district in Uganda (https://www.lshtm.ac.uk/research/centres-projects-groups/general-population-cohort). A total of approximately 1066 GPC participants whose genetics data is available was assessed for current and life-time diagnoses of major depressive disorder, generalized anxiety disorder, suicidality and alcohol and substance abuse.

### Clinical investigations

A questionnaire was administered by trained psychiatric nurses onto a random sample of consenting GPC participants. The questionnaire contained modules for MDD, suicidality, PTSD, alcohol abuse and GAD from the diagnostic and statistical manual for mental disorders edition 4 (DSM-IV) - referenced Mini International Neuropsychiatric Interview (MINI) version 5.0.0 [49]. We had previously translated these modules into Luganda (the language spoken by most of the study participants) [50]. The questionnaire also contained a module for the adult attention-deficit hyperactivity disorder self-report scale (version v1.1) symptoms checklist (https://www.hcp.med.harvard.edu/ncs/ftpdir/adhd/18Q_ASRS_English.pdf). We translated this checklist into Luganda and then used it to assess for traits of ADHD among the study participants.

### Ethical considerations

This study was conducted in compliance with the Code of Ethics of the World Medical Association (Declaration of Helsinki). Ethical and scientific clearance for this study was obtained from the science and ethics committee of the Uganda Virus Research Institute Science and Ethics Committee in August 2022 (Ref# GC/127/916), the Uganda National Council of Science and Technology (Ref# SS1404ES) and the Observational / Interventions Research Ethics Committee of London School of Hygiene and Tropical Medicine (Ref# 28167). Eligible GPC participants (GPC participants whose genetics data is available) were approached and informed about the study by psychiatric research nurses. Written informed consent was obtained from all eligible participants. Consented participants were assessed for mental illnesses, ADHD and alcohol and substance abuse by psychiatric research nurses. Participants who were found to have serious mental illnesses were referred to the mental health clinic at the MRC/UVRI and LSHTM Uganda Research Unit facility at Kyamuliibwa. As per the GPC protocol, each participant was given a bar of soap as compensation for their time.

### Data management

STATA version 17.0 was used for all statistical analyses. Frequencies of socio-demographic characteristics (gender, age, education level, marital status, body mass index) were described using frequencies and percentages for the categorical variables and median (inter-quartile range) for the continuous variables. The prevalence of dichotomised outcome variables (Current MDD, Lifetime MDD, Suicidality, PTSD, Alcohol abuse, GAD and ADHD) were calculated with 95% confidence intervals. Proportions of individuals with more than one mental disorder were estimated. Spearman’s rank correlation was used to assess for inter-item correlation coefficients between the outcome variables. Multivariate logistic regression models were used to evaluate the relationships between each of the outcome variables and their associated factors adjusting for age and sex as apriori confounders. The likelihood ratio approach was used to determine the best fit for the final model. A two-sided P < 0.05 was considered statistically significant. Plausible interactions of socio-demographic factors in the logistic regression models were also investigated across all the investigated mental disorders using a likelihood ratio test.

## Results

The genetic structure of participants of the Uganda Genome Resource has been published elsewhere [51].

The socio-demographic characteristics and clinical variables of the study participants are shown in Table 1.

**Table 1:** Description of the socio-demographic characteristics and clinical variables of the study participants.

Factor	Frequency (%) *n* = 985
Sex	
Male	366 (37.2%)
Female	592 (60.1%)
Missing	27 (2.7%)
Age	
Mean (standard deviation)	40.6 (14.7)
Missing	27 (2.7%)
Ever been married	
Yes	826 (83.9%)
No	132 (13.4%)
Not reported	27 (2.7%)
Age first got married	
Mean (standard deviation)	19.7 (3.9)
Missing	159
Smoking tobacco	
Yes	84 (8.5%)
No	874 (88.7%)
Missing	27 (2.7%)
Consumed alcohol in the last 12 months	
Yes	357 (36.2%)
No	80 (8.1%)
Missing	548 (55.6%)
Ever had high blood pressure	
Yes	76 (7.7%)
No	882 (89.5%)
Missing	27 (2.7%)
Ever had raised blood sugar	
Yes	15 (1.5%)
No	943 (95.7%)
Missing	27 (2.7%)
Body mass index	
Median (interquartile range)	22.1 (19.6, 23.8)

### Prevalence of mental disorders

Life-time MDD was the most prevalent mental disorder (23.3%, 95% CI = 20.7, 25.9) while PTSD was the least prevalent mental disorder (3.1%, 95% CI = 1.8, 5.4). The prevalence of all the mental disorders assessed for are shown in Table 2.

**Table 2:** Prevalence of mental disorders and alcohol abuse among the study participants.

Mental disorder	Prevalence	Prevalence Per 100 (95% CI)
Current MDD	190/984	19.3 (16.9, 21.9)
Life-time MDD	229/984	23.3 (20.7, 25.9)
Suicidality	104/984	10.6 (8.7, 12.6)
PTSD	12/384*	3.1 (1.8, 5.4)
Alcohol abuse	56/984	5.7 (4.4, 7.3)
GAD	127/984	12.9 (10.9, 15.2)
Probable ADHD	91/985	9.2 (7.5, 11.2)

*MDD* Major depressive disorder, *PTSD* Post-traumatic stress disorder, *GAD* Generalised anxiety disorder, *ADHD* Attention-deficit hyperactivity disorder, *CI* Confidence interval. *data collection for PTSD started late hence the lower numbers.

### Correlations among the different mental disorders and alcohol abuse

There was a statistically significant positive correlation between Life-time and current MDD (r = 0.8883, p < 0.05). There was also a statistically significant positive correlation between suicidality and current MDD (r = 0.3532, p < 0.05) and life-time MDD (r = 0.3247, p < 0.05). There was a statistically positive correlations between GAD and current MDD (r = 0.2561, p < 0.05), life-time MDD (r = 0.2311, p < 0.05), suicidality (r = 0.2868, p < 0.05) and PTSD (r = 0.1289, p < 0.05). Table 3 shows the correlation matrix for all the mental disorders and alcohol abuse.

**Table 3:** Correlation matrix for correlations among the different mental disorders and alcohol abuse.

	Life-time MDD	Current MDD	Suicidality	PTSD	Alcohol abuse	GAD	ADHD
Life-time MDD	1.0000						
Current MDD	0.8883*	1.0000					
Suicidality	0.3247*	0.3532*	1.0000				
PTSD	0.0553	0.0694	0.0072	1.0000			
Alcohol abuse	0.0477	0.0993	0.0228	−0.0278	1.0000		
GAD	0.2311*	0.2561*	0.2868*	0.1289*	0.0560	1.0000	
Probable ADHD	−0.0362	0.0251	−0.0357	−0.0268	0.0268	0.0052	1.0000

*MDD* Major depressive disorder, *PTSD* Post-traumatic stress disorder, *GAD* Generalised anxiety disorder, *ADHD* Attention-deficit hyperactivity disorder, *p < 0.05.

### Comorbidity among different mental disorders investigated

**Fig. 1:**
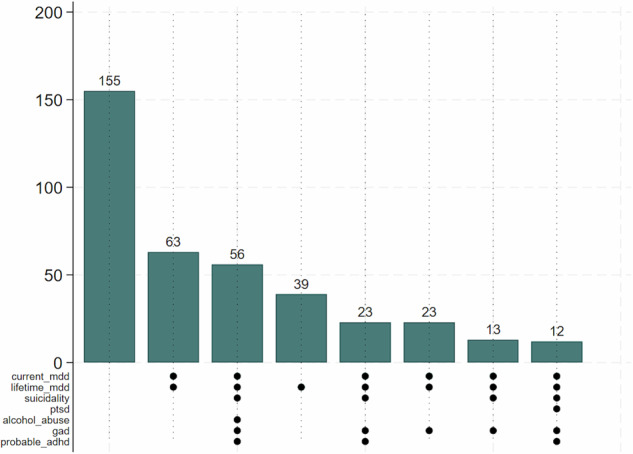
An upset plot showing comorbidity of mental disorders among study participants. Proportions of participants with none, one or more mental disorders are shown, mdd = major depressive disorder, ptsd = posttraumatic stress disorder, gad = generalized anxiety disorder, adhd = attention-deficit hyperactivity disorder.

High comorbidity of mental disorders was observed and these are shown in the upset plot shown in Fig. 1 below. A total of 155 participants did not present with any of the investigated mental disorders, 39 participants had lifetime MDD, 63 participants had both current and lifetime MDD. A total of 23 participants had both current MDD and lifetime MDD alongside with GAD. The comorbidity extends to another 23 participants who in addition to MDD and suicidality had ADHD. A total of 56 participants had both current and lifetime MDD, suicidality, GAD as well as alcohol abuse and probable ADHD. A total of 12 participants had current and lifetime MDD, suicidality, PTSD, GAD and probable ADHD.

### Factors associated with the different mental disorders

There was a statistically significant association between sex and alcohol abuse (AOR = 0.26 [0.14, 0.45], p < 0.001) and GAD (AOR = 1.78 [1.09, 2.49], p = 0.019). Being female was a risk factor associated with GAD while protective against alcohol abuse. BMI was also statistically associated with suicidality (AOR = 0.85 [0.73, 0.99], p = 0.041), alcohol abuse (AOR = 0.86 [0.78, 0.94], p = 0.003) and GAD (AOR = 0.93 [0.87, 0.98], p = 0.008). Per unit increase in BMI was protective against suicidality, alcohol abuse and GAD. There was also a statistically significant association between high blood pressure and life-time MDD (AOR = 2.87 [1.08, 7.66], p = 0.035) and probable ADHD (AOR = 1.99 [1.00, 3.97], p = 0.050). High blood pressure was a risk factor for both life-time MDD and probable ADHD. There was also a statistically significant association between tobacco smoking and alcohol abuse (AOR = 3.2 [1.56, 6.67], p = 0.002). Ever been married was also a risk factor for probable ADHD (AOR = 2.12, [0.88, 5.14], p = 0.049). These results are summarised in Table 4.

**Table 4:** Results of fitting multiple logistic regression models for factors associated with the different mental disorders.

Variable	Level	Adjusted odds ratios (95%CI)				
		Suicidality	Alcohol abuse	GAD	ADHD	Life-time MDD
Sex	Male	1	1	1	1	1
	Female	2.14 (0.77, 5.90)	**0.26 (0.14, 0.45)**^**λ**^	**1.78 (1.09, 2.49)**^**ƒ**^	1.02 (0.65, 1.61)	0.93 (0.53, 1.63)
Age in years	Per year increase	1.01 (0.98, 1.04)	0.99 (0.97, 1.01)	0.99 (0.98, 1.00)	1.00 (0.99, 1.01)	1.00 (0.98, 1.02)
Ever been married	No	1	1	1	1	1
	Yes	1.16 (0.21, 6.46)	2.5 (0.97, 6.45)	1.35 (0.79, 3.01)	**2.12 (0.88, 5.14)**^**ƒ**^	2.13 (0.79, 5.66)
Smoke tobacco	No	1	1	1	1	1
	Yes	2.46 (0.40, 15.07)	**3.20 (1.56, 6.67)**^**γ**^	1.15 (0.52, 2.50)	1.67 (0.79, 3.53)	0.33 (0.09, 1.26)
Drink alcohol	No	1	1	1	1	1
	Yes	1.03 (0.20, 5.21)	**9.01 (1.21, 67.11)**^**ƒ**^	0.79 (0.39, 1.61)	0.69 (0.32, 1.49)	0.96 (0.30, 3.01)
High blood pressure	No	1	1	1	1	1
	Yes	0.39 (0.05, 3.31)	2.10 (0.81, 5.39)	0.82 (0.37, 1.78)	**1.99 (1.00, 3.97)**^**ƒ**^	**2.87 (1.08, 7.66)**^**ƒ**^
HIV status	Negative	-	1	1	1	1
	Positive	-	0.92 (0.32, 2.65)	1.42 (0.76, 2.68)	1.36 (0.65, 2.83)	1.87 (0.73, 4.77)
Raised blood sugar	No	1	-	1	1	1
	Positive	1.53 (0.38, 6.06)	-	0.60 (0.08, 4.67)	0.68 (0.09, 5.30)	1.98 (0.38, 10.44)
Age first got married	Per year increase	0.95 (0.89, 1.02)	1.00 (0.92, 1.09)	0.99 (0.94, 1.06)	1.03 (0.96, 1.10)	-
Body mass index	Per unit increase	**0.85 (0.73, 0.99)**^**ƒ**^	**0.86 (0.78, 0.94)**^**γ**^	**0.93 (0.87, 0.98)**^**γ**^	0.96 (0.89, 1.02)	1.00 (0.93, 1.08)

*GAD* generalized anxiety disorder, *ADHD* attention-deficit hyperactivity disorder, *MDD* major depressive disorder, *HIV* Human immune-deficiency syndrome, - variables omitted due to perfect collinearity, ƒ = p < 0.01, γ = p < 0.01, λ = p < 0.001.

Bolded figures show significant associations.

There was no statistically significant association between any of the investigated factors and PTSD and current MDD (please see supplementary material, Table S1). Also, there were nonsignificant interactions among the examined socio-demographic factors across all the investigated mental disorders (please see supplementary material, Table S2) and thus we did not report adjusted effect sizes after any interaction.

## Discussions

In this study, we determined the prevalence and correlates of MDD, suicidality, GAD, PTSD, probable ADHD and alcohol abuse among participants of the GPC of MRC/UVRI and LSHTM Uganda research Unit, who contributed genetics data to the Uganda Genome Resource.

We have observed prevalence of 19.3 and 23.3% for current and life-time MDD respectively. This prevalence is more than the global prevalence of 3.4% which has been reported for depressive disorders [1]. This prevalence is also more than the prevalence of 4.5% which has been reported in SSA for depressive disorders [1]. However, this prevalence is within the prevalence estimates of 4.2 – 29.3% which have been reported among general populations from various study sites in Uganda [7–11]. Higher rates of MDD in Uganda could be due to poverty, ecological factors and social deprivation (no formal education, unemployment, broken family, low socioeconomic status, food insecurity) [7–9, 11].

The observed prevalence of 3.1% for PTSD is less than the prevalence of 11.8% which has been reported among a post-war area in three districts of Northern Uganda [52]. The observed lower prevalence could be due to the fact that this community has not experienced any war or major natural traumatic event since the Ugandan civil war which ended in 1986. It is also worthy of note that the observed prevalence is also comparable to a cross-national lifetime prevalence of 3.9% which was reported by the World mental health survey [53].

The observed prevalence of 10.6% for suicidality is within range of the 1–55% and 0.6–14% prevalence for suicidal ideation and attempted suicide respectively which were observed by a systematic review and meta-analysis among a general population in Ethiopia [54]. This prevalence is also comparable to a prevalence of 12.1% which was reported among randomly selected individuals from three districts of Uganda [55].

The observed prevalence of 5.7% for alcohol abuse is less than the average population lifetime prevalence of 10.7% which was reported by the World mental health survey [56] and the prevalence of 9.8% which was reported among adult populations in Uganda [57].

The observed prevalence of 12.9% for GAD is slightly higher the lifetime prevalence of 9% which has been reported for the United States [58] and the 9.1% prevalence which has been observed among people living with the human immunodeficiency virus (HIV) [59]. However, a prevalence as high as 33.2% has been reported among HIV patients in a tertiary institution in Nigeria [60].

We have observed a prevalence of 9.2% for probable ADHD. This prevalence is more than the global prevalence of 1.1% which has been reported for ADHD [1]. This prevalence is also more than the prevalence of 0.6% which has been reported in SSA [1]. The big discrepancy between the observed prevalence and that reported by previous studies could be due to the fact that the assessment tool used by this study assesses for ADHD symptoms without considering functional impairment, thus we report the disorder as probable ADHD. It is however worthy of note that no study has previously determined the prevalence of ADHD among adult Ugandans from a general population. It is also worthy of note that the observed prevalence is comparable to a prevalence of 11% which has been reported among Ugandan children attending paediatric neurology and psychiatry clinics at Mulago Hospital [61] and is less than the prevalence of 40.9% which has been reported among adult Ugandans with substance use disorder attending the Butabika Hospital [62].

Sex was significantly associated with both alcohol abuse and GAD. Female sex was protective against alcohol abuse, a finding which is consistent with findings from previous studies in Uganda and the rest of the world, where alcohol use was reported to be higher among men than women [57, 63, 64]. Being female was however associated with increased odds of GAD. This finding is in line with previous studies which have reported women to be 2- to 3- times more likely to meet a lifetime criterion for GAD as compared with men [65–67]. Increased risk for GAD among females could be due to fluctuations in levels of progesterone and oestrogen across the lifespan [68].

Body mass index was associated with suicidality, alcohol abuse and GAD. A unit increase in BMI was protective against suicidality. This finding is consistent with findings from a systematic review and meta-analysis which has reported obesity and overweight to be protective against attempted suicide and suicide mortality [69]. However, definition of suicidality and sex need to be accounted for when interpreting associations between BMI and suicidality. For example, a positive association between obesity and overweight with suicidal ideation has been reported [69] and BMI has been reported to be protective against suicidality among men and paradoxically a risk factor for suicidality among women [70]. A unit increase in BMI was also protective against alcohol abuse. This finding is also consistent with results from a longitudinal study which reported that across adolescence, obesity was protective against alcohol-related problems during early adulthood [71]. This finding supports the food-alcohol competition hypothesis that a tendency to consume processed or sweet high-fat foods compete with a tendency to drink alcohol [72]. A unit increase in BMI was also protective against GAD. This finding is also in agreement with findings from a previous study which reported a negative correlation between BMI and GAD among university students in Bahrain [73]. However this finding contradicts findings from other studies which have reported BMI as a risk factor for GAD [74, 75] and more studies will be required to elucidate this.

High blood pressure was significantly associated with life-time MDD and ADHD. High blood pressure was associated with increased odds of life-time MDD. This finding is in line with a large systematic review and meta-analysis which reported MDD to be a risk factor high blood pressure [76]. This association has been demonstrated to be bidirectional by a large prospective study among young and middle-aged adults [77]. Depressive symptoms were found to be associated with incident hypertension and higher blood pressure levels to be associated with a decreased risk for developing depressive symptoms [76, 77]. Mechanisms that underlie the association between high blood pressure and MDD are yet to be elucidated. High blood pressure was also a risk factor for ADHD. This finding is also in line with findings from a large prospective Swedish cohort study which reported ADHD to be associated with high risk for developing hypertension [78]. It has been proposed that ADHD-associated deficits in delayed discounting and reinforcement sensitivity could impair the future-oriented activities needed to promote good health and thus lead to the development of metabolic disorders like hypertension and type-2 diabetes mellitus [78–80].

Smoking tobacco was a risk factor for alcohol abuse. This is consistent with findings from a previous multi-site study which has reported a high correlation between alcohol consumption and tobacco use in Uganda [81] and sub-Saharan Africa in general [82].

Ever been married was significantly associated with ADHD. Individuals who had ever been married had higher odds of being a case of ADHD. This finding could be due to the fact that ADHD often leads to termination of marriages when it is not recognised and treated properly [83, 84].

Past MDD is a predictor of current (recurrent MDD), thus the significant positive correlation observed between current and past MDD. This is due to the high relapse rates which have been reported in MDD [85]. The high relapse rates could be due to variability in response to treatments. A recent meta-analysis of 87 randomized clinical trials (N = 17,540) has indeed reported 14% more variability in response to antidepressants as compared to placebo [86]. Differences among patients or biological heterogeneity within MDD have been proposed as mechanisms which could explain this variability [86, 87]. The significant positive association between suicidality and MDD is due to the high prevalence of suicidality which has been reported in MDD [88]. The significant positive association between GAD and MDD, suicidality and PTSD respectively is also due to the high prevalence of GAD in these disorders. For example, prevalence of GAD as high as 71.7% in MDD [89], 51.9% in suicidality [90] and 27.6% in PTSD [91] have been reported.

Mental disorders investigated exhibited a high degree of comorbidity. The high comorbidity among mental disorders has been attributed in part due to a high level of common genetic risk factors as well as environmental risk modifiers which are shared across several mental disorders [92].

Since common mental disorders are quite prevalent in the Uganda genome resource, there is opportunity to collect the phenotype data and link it with the existing genetics data to conduct genome-wide association studies for several mental disorders. The Uganda genome resource is also poised to grow in sample size and will include proteomics, metabolomics, and single-cell genomic studies which can advance functional genomics research for mental conditions in Uganda and Africa at large. Also, given the longitudinal design of the GPC, there is opportunity to track changes and understand the dynamics of mental disorders and their genetic underpinnings and can ultimately provide insights into causality and progression of mental disorders.

## Limitations

We could not elucidate the causation of mental disorders due to the cross-sectional nature of the study. Also, in data collection, we relied on the respondents’ memory and recall bias which may potentially result in a systematic bias against the recall of temporally distant events. Also, given that this was a volunteer-based population study, we may not have captured the most severely affected individuals and there is a likelihood of underrepresentation of the most disadvantaged people in the community studied. Also, while our sample of 985 participants allowed for preliminary insights into the prevalence and correlates of common mental disorders among participants of the Uganda genome resource, we recognize the need for a larger cohort to enhance statistical power and robustness of our findings, most particularly for disorders with a lower prevalence such as PTSD. Despite this limitation, this study makes a great contribution given the dearth of similar studies in Africa. However, we are already undertaking a study in the GPC which seeks to gather phenotype data and biological samples on various mental health conditions among participants of the Uganda genome resource who are still alive and present in the GPC (n = 4000) and additional data from 6,000 GPC participants. Collectively, this effort will encompass 10,000 participants; all of these will have their DNA sequenced using the blended genome-exome technology at the Broad Institute in the United States.

## Conclusions

Common mental disorders are quite prevalent among people who comprise the Uganda Genome Resource with current MDD being the most prevalent disorder and PTSD being the least prevalent disorder.

As participants of the Uganda Genome Resource were drawn from an open general population cohort and can still be traced, there is opportunity for psychiatric genetics research where participants can be traced, assessed for mental disorders and the mental disorders data can then be linked with the existing genetics data to allow performance of psychiatric genetics studies. There is currently no representation of people from Africa in global psychiatric genetics databases and performing genetics studies in this cohort will contribute to efforts towards addressing the underrepresentation of people from Africa in global psychiatric genetics databases while provide insights into the genetic nature of mental disorders in Africa.

## Supplementary information

Supplementary material S1

Supplementary material S2

## Data Availability

The epidemiological data for this study is available upon request from the principal investigator, following the MRC/UVRI and LSHTM Uganda Research Unit’s data sharing policy which can be found at: https://apps.mrcuganda.org/mrcdatavisibility/Home/. For the genetics data, the array and low- and high-depth sequence data were deposited at the European Genome-phenome Archive (EGA, https://www.ebi.ac.uk/ega/, accession numbers EGAS00001001558/EGAD00010000965, EGAS00001000545/EGAD00001001639, and EGAS00001000545/EGAD00001005346, respectively. Requests for access to the genetics data may be directed to UGR@mrcuganda.org.
