# Supplementary material for: Prevalence and correlates of common mental disorders among participants of the Uganda Genome Resource: Opportunities for psychiatric genetics research
Source: Mol Psychiatry. 2024 Jul 14;30(1):122–30. doi: 10.1038/s41380-024-02665-8 (PMC11649557; doi:10.1038/s41380-024-02665-8)
Supplement: Supplementary file 1 — Supplementary material S1 [file 41380_2024_2665_MOESM1_ESM.docx]

**Supplementary material S1**

| Factor | Level | Current MDD  Adjusted Odds Ratios (95%CI) | P value | PTSD  Adjusted Odds Ratios (95%CI) | P value |
| --- | --- | --- | --- | --- | --- |
| Sex | Male | 1 |  | 1 |  |
|  | Female | 1.29 (0.84, 1.77) | 0.211 | 0.54 (0.16, 1.78) | 0.313 |
| Age in years | Per year increase | 1.03 (0.99, 1.02) | 0.102 | 0.99 (0.95, 1.03) | 0.578 |
| Ever been married | No | 1 |  | 1 |  |
|  | Yes | 1.37 (0.71, 2.27) | 0.411 | 1.42 (0.19, 7.68) | 0.843 |
| Smoke tobacco | No | 1 |  | 1 |  |
|  | Yes | 1.22 (0.67, 2.24) | 0.521 | 1.22 (0.12, 12.07) | 0.860 |
| Drink alcohol | No | 1 |  | - |  |
|  | Yes | 1.21 (0.61, 2.08) | 0.677 | - |  |
| High blood pressure | No | 1 |  | 1 |  |
|  | Yes | 1.51 (0.65, 2.03) | 0.635 | 2.18 (0.23, 20.96) | 0.500 |
| Raised blood sugar | No | 1 |  | - |  |
|  | Yes | 0.99 (0.27, 3.60) | 0.992 | - |  |
| HIV status | Negative | 1 |  | - |  |
|  | Positive | 1.20 (0.68, 2.12) | 0.422 | - |  |
| Age first got married | Per year increase | 0.97 (0.91, 1.01) | 0.103 | 1.15 (0.99, 1.34) | 0.075 |
| Body mass index | Per unit increase | 0.97 (0.93, 1.02) | 0.229 | 1.07 (0.95, 1.20) | 0.279 |

***Table S1:*** *Results of fitting multiple logistic regression models for association of different factors with post-traumatic stress disorder and current major depressive disorder.* MDD = major depressive disorder, PTSD = post-traumatic stress disorder.
