# Supplementary material for: Prevalence and correlates of common mental disorders among participants of the Uganda Genome Resource: Opportunities for psychiatric genetics research
Source: Mol Psychiatry. 2024 Jul 14;30(1):122–30. doi: 10.1038/s41380-024-02665-8 (PMC11649557; doi:10.1038/s41380-024-02665-8)
Supplement: Supplementary file 2 — Supplementary material S2 [file 41380_2024_2665_MOESM2_ESM.docx]

**Supplementary material S2**

| **Interaction terms** | **AOR 95%CI** | ***p-value*** |
| --- | --- | --- |
| Sex~age | 1.03(0.87;1.61) | 0.453 |
| Educational level~sex | 1.23(0.61;1.75) | 0.315 |
| Educational level~age | 0.99(0.71;1.23) | 0.754 |
| Sex~SEI | 0.89(0.69;1.17) | 0.644 |
| Educational level~SEI | 1.11(0.93; 1.97) | 0.328 |
| Age~SEI | 1.31(0.91;2.01) | 0.234 |

***Table S2:*** *Interaction of socio-demographic factors across various mental disorders investigated. AOR = adjusted odds ratio, CI = confidence interval, SEI = socio-economic index.*
